# Risk of needle tract seeding after coaxial ultrasound-guided percutaneous biopsy for primary and metastatic tumors of the liver: report of a single institution

**DOI:** 10.1007/s00261-019-02120-1

**Published:** 2019-07-05

**Authors:** Dagmar Schaffler-Schaden, Theresa Birsak, Ramona Zintl, Barbara Lorber, Gottfried Schaffler

**Affiliations:** 1 Institute of General Practice, Family Medicine and Preventive Medicine, Paracelsus Medical University, Strubergasse 21, 5020 Salzburg, Austria; 2 Department of Radiology and Nuclear Medicine, Hospital of St. John of God, Kajetanerplatz 1, 5020 Salzburg, Austria; 3 Faculty of Natural Sciences, University of Salzburg, Hellbrunner Strasse 34, 5020 Salzburg, Austria; 4 Paracelsus Medical University, Strubergasse 21, 5020 Salzburg, Austria

**Keywords:** Coaxial biopsy, Needle tract seeding, Liver biopsy, Ultrasound-guided biopsy

## Abstract

**Purpose:**

The objective of this study was to determine the incidence of needle track seeding after ultrasound-guided percutaneous biopsy of indeterminate liver lesions with a coaxial biopsy system without any other additional intervention or ablation therapy.

**Methods:**

We identified 172 patients in a retrospective cohort study who underwent ultrasound-guided biopsy due to a liver mass in our institution between 2007 and 2016. The same coaxial biopsy system was used in all patients, no consecutive ablation was performed.

**Results:**

None of the finally included 131 patients developed neoplastic seeding. There was one major complication (0.76%), the rest of the complications were minor (3.8%) and did not require further intervention.

**Conclusion:**

Needle track seeding is a rare delayed complication after percutaneous liver biopsy. Coaxial liver biopsy is a safe method to obtain multiple samples with a single punch in patients with primary or metastatic liver lesions.

## Background

There is increasing demand for pathologic specimens in modern medicine that is partly being driven by personalized medicine. Ultrasound-guided percutaneous needle biopsy is still the method of choice for the assessment of focal liver lesions with suspected malignancy due to several advantages as lack of radiation exposure, low cost, and direct visualization of the needle position in real time [1]. Complications of percutaneous liver biopsy are uncommon, but may encompass bleeding, hematoma, infection, pneumothorax, or perforation [2]. A safe ultrasound-guided biopsy requires a normal coagulation status, an accessible target, and a cooperative patient [3]. One rare, but serious complication after percutaneous liver biopsy is needle tract seeding, which is a concern particularly in liver transplant recipients. The insertion of the needle and biopsy of a malignant lesion can cause spreading of tumor cells along the needle track. Usually, seeding after liver biopsy is defined as nodular neoplastic tissue along the needle tract outside the liver capsule appearing in the peritoneal cavity, the subcutaneous tissue, the abdominal muscles, or the skin. The reported incidence of seeding after ultrasound-guided liver biopsy shows wide variation depending on the technique used, the study population, and the duration and quality of surveillance of the follow-up. Seeding rates after liver biopsy have been reported within a range from 0% up to 19% [4, 5]. Presumed risk factors for needle track seeding are aggressiveness and location of the tumor, patients’ immunosuppression, the size of the needle, and the number of needle passes. While most studies report needle tract seeding after biopsy of hepatocellular carcinoma (HCC), information about biopsies of metastatic lesions of the liver is very scarce [5, 6]. Several authors suggested an increased risk of neoplastic seeding in HCC when ablation techniques were combined with biopsy and recommended to avoid biopsy whenever possible. Increased risk of seeding was also reported when Radiofrequency ablation (RFA) followed biopsy, even when track ablation has been performed [7–9]. The time interval between the biopsy and the discovery of the neoplastic seeding also varies greatly due to the quality and duration of patient follow-up. Treatment of choice for needle track seeding is radical surgical excision, local radiotherapy, or high-intensity-focused ultrasound [8].

To minimize the risk of potential needle seeding, coaxial biopsy was considered a safe method compared to other techniques [4, 10] as it is possible to obtain multiple samples of the lesion with a single puncture [11]. Hence, the number of needle passes is reduced and the biopsy is harvested in a closed system which lowers the risk of losing sample tissue. However, evidence about the prevalence and safety of coaxial liver biopsy particularly in patients with metastatic liver disease without consecutive ablation is still lacking.

The aim of this study was to evaluate the risk of needle seeding and complications in a cohort after liver biopsy without any additional ablation using a coaxial needle system in a single center.

## Methods

**Fig. 1:**
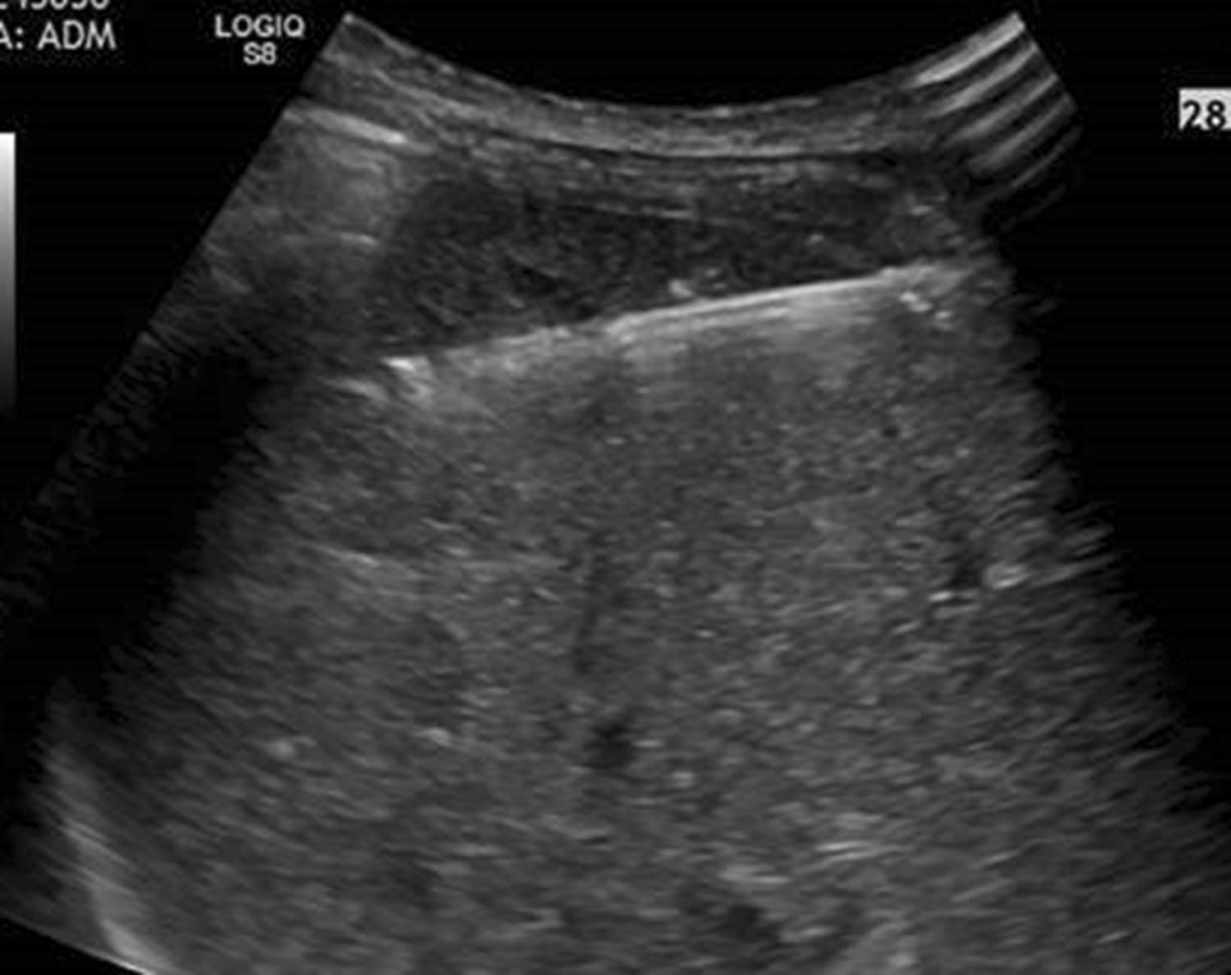
Biopsy with coaxial technique in a 77-year-old female patient with multifocal lesions

Data of 172 patients, who underwent an ultrasound-guided biopsy of the liver with a coaxial needle system in the years 2007–2016 in the department of radiology of the St. John of God Hospital in Salzburg, were evaluated. Only patients with malignant lesions were included, patients with benign lesions, RFA, or other ablation techniques were excluded. Characteristics of liver lesions as size and distribution were recorded as well as complications. Lesions were subdivided into unifocal, multifocal, and diffuse. Diameter of lesions was assessed by measuring the largest in multifocal and diffuse lesions (Fig. 1).

Minimum follow-up was > 30 days after biopsy. All biopsies were performed by radiologists in a single institution with high expertise in sonography-guided biopsy.

Patient data were retrieved from electronic medical records. Platelet count was confirmed ≥ 50,000/µL and an international normalized ratio (INR) ≤ 1.5 at time of biopsy was obligatory. All patients fasted 6 h before biopsy, no antibiotics were used. Four patients had ultrasound-guided paracentesis due to ascites before biopsy. All biopsies were performed under local anesthesia using freehand technique. Nine patients received additional sedation with midazolam. The ultrasonic devices Toshiba Aplio I800, Siemens Acuson Antares, GE Logiq S8 and GE Logiq S8 R3 were used for examination.

For all patients an automatic biopsy system (True-Core TM II, Argon Medical Devices) with a 17G introducer and a coaxial needle (18G) matching the system was used. The biopsy technique adopted for our use was originally described by Lindgren [12]. However, for improved safety and convenience, two out of seven radiologists (3–30 years of experience) work closely together during each liver biopsy. Initially, detailed ultrasound scanning is always performed to determine the path of the biopsy. For lesions in both hepatic lobes, a subcostal approach is preferred. Lesions that are not accessible in this way are biopsied using an intercostal approach; attention is paid not to injure the diaphragm and the adjacent pleura.

One radiologist performs a real-time scan, while the other radiologist marks the entry point for the biopsy under aseptic conditions. The biopsy track is infiltrated using Mepivacaine Hydrochloride 2% to the peritoneum. The introducer is inserted transcutaneously and the tip of the introducer is advanced under continuous ultrasound guidance to the edge of the mass using freehand technique. The track is controlled sonographically during biopsy to ensure precise sampling of the target lesion and to avoid laceration of major vessels. After positioning the introducer’s tip at the margin of the lesion, the automatic firing device provides a spring-loaded advancement of the stylet further into the lesion followed by a subsequent advancement of the cutting needle over the stylet. Tissue protruding into the side notch is cut off by advancement of the cutting needle and entrapped. This technique preserves the tissue architecture for further histopathological examinations. While the introducer stays in place, the coaxial needle is retracted and the specimen is harvested. After biopsy, patients were asked to rest in bed for 4 h. All patients underwent ultrasound control routinely 4 h after biopsy for early detection of potential complications. Potential seeding was defined as neoplastic tissue along the needle tract. Follow-up was performed with an imaging procedure (Sonography in all patients, CT or MRT possibly in addition) by the radiologists according to oncologic guidelines for the respective underlying disease.

This study was approved by the Ethics Committee of Salzburg (ID 415-E/2450/2-2019). All patients gave written informed consent prior to biopsy.

## Results

The final study group encompassed 131 persons. 11 persons were excluded due to incomplete data, 30 persons had a follow-up ≤ 30 days and were excluded from the final analysis. Characteristics of the study group and liver lesions are available in Table 1. The main complication reported was bleeding (4.6%). There was one case of death among all patients following severe hemorrhage. This woman had multiple liver metastases due to lung cancer and refused blood transfusion and further intensive care treatment. The remaining 5 patients with bleeding were treated with compression only, there was no major complication requiring therapeutic intervention (e.g., blood transfusion, embolization, drainage, or surgery).

**Table 1:** Demographic data of patients and types of lesions

Characteristics	Data
Sex (m)	67 (51%)
Age	
Range	31–91
Median	69
Mean ± SD	68.1 ± 10.8
BMI (kg/m^2^) (*n* = 116)	
< 18.5	5 (4%)
18.5–24.9	44 (38%)
≥ 25	67 (58%)
Follow-up (days)	
Range	31-3437
Median	315
Mean ± SD	648 ± 784
No. of punches (*n* = 130)	
1	37 (28%)
2	61 (47%)
3	27 (21%)
4	5 (4%)
Pain	24 (18%)
Bleeding	6 (5%)
Primary (HCC + CC)	*n* = 44
Metastatic	*n* = 87
Mean size overall (*n* = 120)	2.1 cm
Multifocal	80/127
Diffuse	13/127

24/131 (18.3%) persons reported pain, none of them had hemato-or pneumothorax or sepsis/abscess. There was no case of needle tract seeding within the follow-up in our cohort. Rate of major bleeding was 0.76% overall, while rate of minor bleeding was 3.8%. No patient experienced pain and bleeding simultaneously. The majority of patients were considered overweight (BMI ≥ 25 kg/m^2^). Only one person with a BMI > 30 kg/m^2^ reported pain. Overall BMI was not associated with bleeding or pain, not even in obese patients. Age was not associated with increased risk of bleeding or pain. Risk of bleeding did not differ in patients with primary hepatic tumors (HCC and CC, cholangiocarcinoma) or with metastatic disease but was elevated in patients with lesions ≥ 2.5 cm (see Table 2). Mean diameter of lesions in patients with postinterventional bleeding was 5.3 cm. Histology confirmed metastasis (*n* = 87), CC (*n* = 19), and HCC (*n* = 25). 80 patients already had multifocal metastatic disease. No difference regarding pain was found related to the underlying disease. The mean follow-up was 21.6 months (range 31 days–114.5 months). In the majority (47%) of patients 2 samples were obtained (range 1–4 cores). No association was found between the number of cores taken and the occurrence of bleeding/pain.

**Table 2:** Risk ratio of the main risk factors

Risk Factor	*n* (%)	RR pain	RR bleeding
Female	64 (49)	1.24	1.05
Metastatic disease	87 (66)	1.23	1.01
Multifocal findings (*n* = 127)	80 (63)	1.53	1.70
Lesion: ø ≥ 2.5 cm (*n* = 120)	76 (63)	1.01	2.32

### Statistics

The primary outcome was the rate of needle tract seeding after percutaneous biopsy for an indeterminate liver lesion, the secondary outcome was the rate of complications.

Means, standard deviations, ranges, and absolute and relative frequencies were used as descriptive statistics for patient characteristics. Risk factors (sex, age, primary/metastatic disease, uni-/multifocal findings, size of lesion) were analyzed with Risk Ratio. R software (R version 3.5.0, R Core Team) was used for the statistical analysis of the data.

## Discussion

This study summarizes the results of 131 patients with primary or metastatic malignant liver lesions, who underwent a coaxial needle biopsy in a single department without additional ablation therapy. Age, BMI, sex, underlying disease, and number of cores were not associated with post-biopsy complications. None of these patients developed needle track seeding. Although the rate of minor bleedings requiring no additional intervention in our cohort seems rather high (3.8%), we believe that this is attributed to consistent post-biopsy monitoring in our institution, because these patients were clinically inconspicuous and would not have been detected otherwise. One earlier study including 101 patients with HCC reported zero needle tract seeding with the coaxial biopsy technique as well, although 34 of these had consecutive RFA. Bleeding complications were slightly higher and more severe compared to our results (6.25%, 5 patients requiring transfusion and three of these underwent angiographic embolization) [4]. In a large sample of 1060 patients undergoing renal and hepatic biopsies, the coaxial and non-coaxial techniques were considered equivalent in terms of complications. The incidence of needle seeding was not reported in this study [13]. On the contrary, two RCTs comparing the coaxial and non-coaxial method in perineal prostate biopsy and biopsy of renal parenchyma confirmed a lower complication rate for the coaxial biopsy system [14, 15].

The incidence of needle tract seeding was reported in two reviews after biopsy of HCC with 2.29% [7] and 2.7% [16], respectively. These series included CT-guided biopsies and different biopsy techniques. Most studies refer to patients with HCC. Literature about needle tract seeding after biopsy of liver metastasis is rare and often limited to case reports [17]. Two older studies reported a very high incidence of needle seeding after liver biopsy of colorectal metastasis (16% versus 19% respectively), which encouraged the authors’ assumption that these patients have a higher risk for neoplastic seeding [5, 6]. A more recent study including patients with colorectal and breast cancer liver metastasis observed a seeding rate of 1% after biopsy in the colorectal cancer group and a rate of 6% when other interventions were added. All biopsies in this study were performed as a fine needle aspiration biopsy or a core needle biopsy [18].

Risk of needle seeding was observed even when the coaxial technique was used. One study reported a case of seeding 5 years after CT-guided coaxial biopsy of HCC [19].

A number of authors refer to an increased rate of needle seeding when biopsy and ablation of focal liver lesions are combined [7, 9, 20, 21]. Szpakowski et al. recently reported an overall seeding rate of 1.36% (6/441) after HCC biopsy. The rate of needle seeding after biopsy only was significantly lower than with consecutive ablation (0.74% vs. 2.33%). One of the 6 patients with seeding had biopsy with a coaxial system, but this patient underwent RFA in the same session. Abdominal wall metastasis occurred 33 months later [22]. Although the impact of biopsy and/or ablation on the risk of seeding is not clear yet, some authors recommend to avoid biopsy when ablation is intended. Other authors did not confirm an increased risk of seeding when biopsy for HCC was performed before RFA [23]. Interpretation of study results is difficult due to heterogeneous techniques and study populations, but it is presumed that the tract for the biopsy needle is usually not the same as for RFA. Many studies do not distinguish between biopsy only and/or additional ablation techniques, which hampers comparability.

The mean time until the detection of needle tract seeding varies in the published studies between 6 months and 5 or even 6 years [18, 19, 24]. These data indicate that seeding can occur with a delay of several years and this enhances the importance of adequate follow-up. The quality and length of follow-up certainly plays a key role in the detection of neoplastic seeding. The method of follow-up is often not reported or patients received only physical examination without any imaging [4]. Mean follow-up varies between 13.6 months and 34.3 months, respectively [4, 8]. Although the majority of our patients already had metastatic disease, the mean follow-up in our cohort was 21.5 months. Fortunately, in most cases seeding does not seem to affect patients’ long-term survival when treated accurately, this was also confirmed for needle tract seeding after biopsy of liver metastasis [10, 18].

Our study is one of the larger series of malignant liver lesions strictly biopsied with a coaxial system without any other intervention or ablation. The presumption that risk of needle seeding is much higher in patients with metastatic disease was not confirmed in our study which encompassed 44 cases with primary hepatic disease (HCC + CC) and 87 patients with metastatic disease [6].

One limitation of the study is the retrospective design, although patients were carefully selected. Individual pain levels and overall (long term) survival were not analyzed.

Strengths of this study are the use of a homogeneous biopsy technique with a well-documented adequate follow-up from a single institution. All patients underwent the same procedure with the same equipment and had follow-up with at least one imaging technique. Importantly, this study includes patients with metastatic disease since evidence about needle tract seeding in this group of patients is very scarce.

## Conclusion

The study confirms the relevance and safety of ultrasound-directed coaxial biopsy technique in focal liver lesions. Ultrasound-guided liver biopsy with a coaxial needle system is a technique with a low complication rate in experienced hands and the risk of needle seeding after liver biopsy can be reduced to a minimum. However, adequate length and quality of follow-up is required to identify delayed needle tract seeding.
